# Retrospective Study of the Use of Heparins in Pregnant Women and *in vitro* Testing on the HCT 116 Colorectal Carcinoma Cell Line

**DOI:** 10.2478/jccm-2024-0009

**Published:** 2024-01-30

**Authors:** Felicia Fiat, Diana-Aurora Arnautu, Brenda Cristina Bernad, Alina Anton, Iasmina Marcovici, Alexandra-Denisa Semenescu, Elena Silvia Bernad

**Affiliations:** Department of Obstetrics-Gynecology II, Faculty of Medicine, “Victor Babes” University of Medicine and Pharmacy, Timisoara, Romania; Doctoral School, “Victor Babes” University of Medicine and Pharmacy, Timisoara, Romania; Department of Cardiology, Victor Babeș University of Medicine and Pharmacy, Timisoara, Romania; Center for Neuropsychology and Behavioral Medicine, “Victor Babes” University of Medicine and Pharmacy, Timisoara, Romania; Department of Toxicology, Drug Industry, Management and Legislation, Faculty of Pharmacy, “Victor Babes” University of Medicine and Pharmacy, Timisoara, Romania; Research Center for Pharmaco-Toxicological Evaluations, University of Medicine and Pharmacy Victor Babes, Timisoara, Romania

**Keywords:** heparin, low-molecular-wight heparin, fraxiparine, pregnant women, colorectal cancer, safety profile

## Abstract

**Introduction:**

Pregnant women manifest an increased risk of developing coagulation disorders. Unfractionated heparin (HEP) and low-molecular-weight heparin (LMWHep) are considered as selective medication in the case of pregnancy which needs anticoagulant treatment. In addition to anticoagulant properties, HEP and its derivatives manifest other properties including anti-cancer potential. According to Globocan’s latest data, colorectal cancer (CRC) is the second most encountered form of malignancy in the case of women, manifesting some special particularities, as confusion of symptoms from cancer with symptoms encountered normally in pregnant women (such as constipation or rectal bleeding), delayed diagnosis because of limitations imposed both for the fetus and for the mother, and the need for special treatment.

**Aim:**

The aim of the present work is to follow the incidence and safety of consumption of HEP and LMWHep in the case of pregnant women and to analyze their potential on the HCT 116 colorectal carcinoma cells.

**Results:**

Analyzing the consumption of heparins in case of pregnant women hospitalized from 01.01.2022 to 31.12.2022 at the Pius Brînzeu” Emergency Clinical Hospital from Timisoara, Obstetrics and Gynecology Clinic I, it was observed that 44,6% of the patients were administered the following medication and no administration risks were observed. When tested on HCT 116 cells, heparins manifested a significant anti-migratory effect (with wound healing rates of 2,6%, when tested with HEP 100 UI concentration and 14.52% wound healing rates in case of fraxiparine 100 UI). In addition, different signs of apoptosis were observed, suggesting the pro-apoptotic potential of the tested substances.

**Conclusions:**

Heparins remain the preferred medication to be administered to pregnant women with the potential for coagulation disorders, showing a high safety profile. Testing on the cancerous line of colorectal carcinoma highlights important properties that stimulate future studies, to establish the anti-tumor potential and the exact mechanism of action.

## Introduction

Pregnancy is a special physiological state that increases a woman’s risk of developing thrombosis (five times more than in the case of a non-pregnant woman), which refers to the formation of blood clots in the blood vessels [1]. Heparin (HEP) plays a crucial role in ensuring the health and safety of both the mother and the developing fetus in cases where clotting disorders are a concern. Two common types of HEP are used during pregnancy, unfractionated and low-molecular-weight heparin (LMWHep). LMWHep is often preferred during pregnancy due to its more predictable anticoagulant effect and reduced risk of side effects compared to classic HEP [2].

HEP is one of the most used anticoagulants and antithrombotic, not only in case of pregnant women, discovered at the beginning of the 20th century by William Henry Howell and Jay McLean [3]. The present drug is formulated from animal tissues, mainly from bovine lung and pig intestinal mucosa. Its chemical structure represents a heterogeneous linear glycosaminoglycan substituted with sulfate. In addition to the two main properties, other pharmacological activities, including anti-inflammatory, anti-hypolipidemic, anti-viral, anti-angiogenesis, and anti-tumor potential were confirmed [4]. Based on its main anticoagulant potential, HEP is especially used to avert pulmonary embolism and deep vein thrombosis, as well, the reduction of lipidemia values has also been reported [5,6]. Hyperlipidemia is a frequent metabolic disorder, which significantly contributes to atherosclerosis development, acute pancreatitis risk, and other metabolic sickness [7]. Different studies indicate that HEP and insulin are able to stimulate the lipoprotein lipase activation, thus reducing plasmatic triglyceride values [8,9]. It has the appearance of being an inexpensive, safe, and effective treatment for hypertriglyceridemia associated with acute pancreatitis [9,10].

When about cancer, one of the most common and frightening ailments of today, the link between this and thrombosis is well documented [11,12,13]. Venous thrombus embolism is the most frequently encountered problem of cancer-associated thrombosis (CAT). LMWHep were used for decades as standard treatment for CAT [14,15]. Several data have shown that in addition to cancer-related CAT, HEP or its derivatives are able to impact by manifold ways outright tumor biology through diverse mechanisms, such as angiogenesis, tumor cell signaling, metastasis, and inflammation [4]. Different chemokines, growth factors, and enzymes are amalgamated in these processes, such as P-/L-selectin, heparanase, and CXC chemokine ligand and receptor [16,17,18]. HEP actively interacts with these mediators, implying a cascade of biochemical answers and then upsetting invasion and tumor growth [19,20].

According to Globocan’s latest data, colorectal cancer (CRC) is the second most encountered form of malignancy in the case of women, with an incidence of 16.2/100.000 [21]. The subject of pregnant women with CRC is particular from several points of view: i) confusion of symptomatology from cancer with that encountered normally in pregnant women, such as constipation or rectal bleeding; ii) delayed diagnosis; iii) the need for special therapeutic approaches. Thus, pregnancy may affect the clinical evaluation, presentation, and prognosis of malignancy. Health care providers may delay diagnosis because of the major fetal risks and distraction from potentially significant symptoms and the relatively rare condition encountered in young adults. Colonoscopy is customarily the usual method used to evaluate suspicious colon problems, including cancer. Still, it is not yet an accustomed technique for pregnant women because of the safety issues posed for the fetus. Since the evaluation of CRC usually consist of computed tomography (CT) at the abdominal level to recognize pericolonic extension and metastases, this imagistic method is also not allowed in pregnancy, especially during the first trimester, because of the risk of teratogenic radiation [22].

Emerging from the above, the purpose of this study is to evaluate the consumption and the safety profile of HEP and LMWHep in the case of pregnant women and to analyze their potential on the HCT 116 colorectal carcinoma cells.

## Materials and methods

### Materials

HEP was purchased from Galenika International Kf. (Hungary) and nadroparin (as a model of LMWHep), with the commercial name Fraxiparine (FRAX), from Aspen Notre Dame de Bonneville (France), phosphate buffered saline (PBS), penicillin/streptomycin, and dimethyl sulfoxide (DMSO) were bought from Sigma (Darmstadt, Germany). The Hoechst 33342 reagent was achieved from Invitrogen, MTT (3-(4,5-dimethylthiazol-2-yl)-2,5-diphenyltetrazolium bromide) viability kit from Roche (United Kingdom), fetal bovine serum (FBS) and trypsin-EDTA solution were achieved from Pan Biotech (Aidenbach, Germany). The specific cell culture media, McCoy’s 5A (30-2007) was bought from ATCC (American Type Cell Collection, Poland). All the reagents were used following the manufacturers’ suggestions and presented analytical standard purity.

### Clinical data

The present retrospective study was performed on 92 hospital release notes of patients hospitalized at the „Pius Brînzeu” Emergency Clinical Hospital from Timisoara, Obstetrics and Gynecology Clinic I, between 01.01.2022 and 31.12.2022, that have been analyzed. The following data were collected: age, demographic data, diagnostic, comorbidities, discharge diagnosis, information about the administered anticoagulant treatment (type, name, dose), side effects, the values of the biological parameters (hemoglobin, hematocrit, fibrinogen), type of birth, and the newborns’ Apgar index. The study protocol has been approved by the institutional ethical committee and all included patients having the informed consent signed from the observation sheet, upon hospitalization.

### Cell Culture

For the present study HCT 116 colorectal carcinoma cell line (isolated from the colon of an adult male with colon cancer) was used, which was acquired from ATCC in an individual frozen vial. In order to grow cells, the specific McCoy’s 5A Medium completed with 10% FBS, and the antibiotic mixture (100 U/mL penicillin/100 µg/mL streptomycin) in a concentration of 1%, to avoid microbial contamination, were used. The cell cultures were grown and maintained respecting the favorable conditions, an atmosphere with 5% CO_2_ and a stable temperature of 37 °C, in a humidified incubator.

### Viability Assay

To follow the cellular viability, the MTT test was performed. Summary, HCT 116 cells were grown in 96-well plates, respecting 10^4^ cells/200 µL/well density. After reaching a 80–90% confluence, cells were treated with four concentrations of HEP and FRAX (10, 25, 50, and 100 UI). Attending 72 h of incubation, 10 µL per well of MTT solution (kit I −5 mg/mL) was added and the plate was putted for 3 h in an incubator, the period in which the formazan crystals were formed, and were dissolved after with 100 µL per well of solubilization buffer (kit II), for 30 min, out of the light. In the end, Cytation 5 microplate reader (BioTek Instruments Inc., Winooski, VT, USA) was used to measure at 570 nm the reduced MTT. All experiments were performed three time independently.

### Cell Morphology and Confluence Evaluation

As means of observing changes induced by HEP and FRAX on confluence and morphology, HCT 116 cells were microscopically observed under bright field illumination after 72h of treatment, using the inverted microscope Olympus IX73 (Olympus Corporation, Japan) equipped with DP74 camera. Analysis Software Microplate Gen5 (BioTek Instruments Inc., Winooski, VT, USA) was used to process the pictures.

### Nuclear Morphology Evaluation

Information regarding the type of cellular death executed by HEP and FRAX was obtainable through the utilization of immunofluorescence. The putt-on Hoechst protocol respected the manufacturer’s recommendations. Thus, HCT 116 cells were cultured in 12-well plates, at 1 × 10^5^ cells per well. After observing a confluence of roughly 80%, the cells were stimulated with four different concentrations of HEP or FRAX. 72 h after treatment, the growing medium was replaced with 100 μL per well of staining solution (diluted 1:2000 in PBS). After 10 min of incubation in the dark, at room temperature, the Hoechst reagent was drawn out and cells were triple washed with PBS. The fixed nuclei were imaged with Cytation 1 (BioTek Instruments Inc., Winooski, VT, USA) and pictures were processed using the specific Gen5 Software (BioTek Instruments Inc., Winooski, VT, USA).

### Wound Healing Assay

To identify the capacity of the tested substances to interfere with the migration of HCT 116 cells, a scratch assay was performed, following the indications described in the protocol [23]. Briefly, 2 × 10^5^ cells per well were seeded in 12-well plates, following a 90% confluence, an automatic scratch was drawn using the AutoScratch™ Wound Making Tool provided by BioTek^®^ Instruments Inc. (Winooski, VT, United States). The untied cells were washed with PBS and were removed. The next step, cells were treated with 100 UI of both unfractionated and LMWHep. Photos were taken at two intervals, 0 h and 24 h, by means of the inverted microscope Olympus IX73 provided with DP74 camera (Olympus Corporation, Japan). The wound widths were measured with the software Gen5 ™ Microplate Data Collection and Analysis (BioTek^®^ Instruments Inc., Winooski, VT, United States). The following formula was used to determine the scratch closure rate (%)[23]:

$$\text{Scratch}\;\text{closure}\;\text{rate}=\left[\left(\text{At}0-\text{At}\right)/\text{At}0\right]\times 100$$

where:

At0—scratch closure at initial time 0;
At—scratch closure after 24 hours.

### Statistical analysis

All the in vitro experimental results are expressed as averages ± standard deviation (SD). The statistical obtained differences were compared by one-way ANOVA analysis followed by Dunett’s multiple comparisons post-test. GraphPad Prism software, version 9.4.0 (GraphPad Software, San Diego, CA, USA) was used to perform graphics. The statistically significant variations between obtained results were pointed with * (* p < 0.1; **** p < 0.0001).

## Results

### Demographic data

**Fig. 1.**
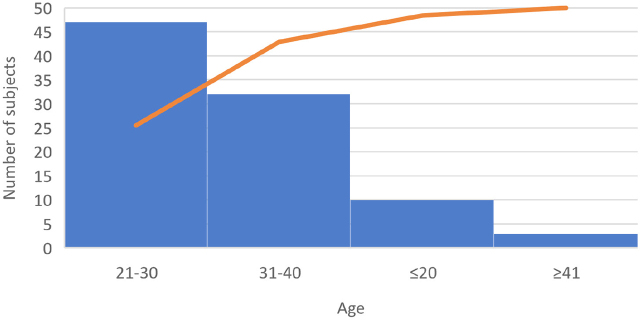
Age distribution of the subjects

**Fig. 2.**
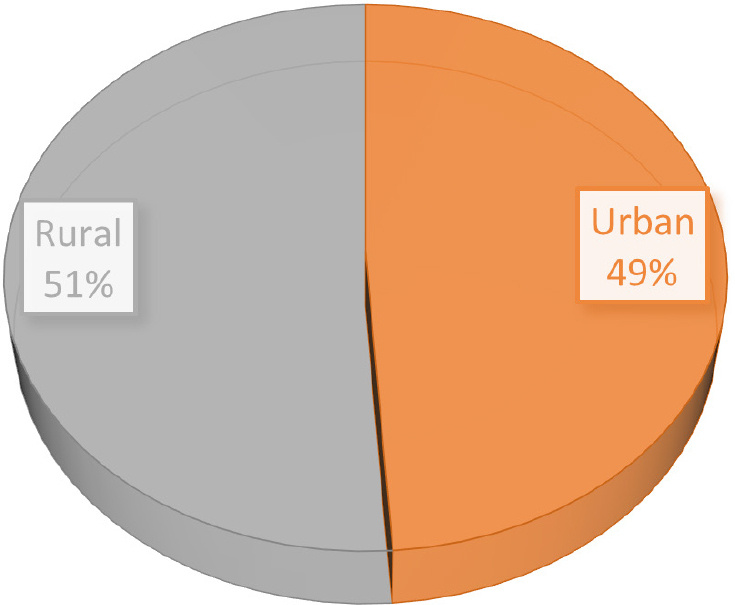
Rural/urban area distribution of subjects

The present study aimed to follow causes and effect of HEP and its derivates administration in case of pregnant women. 92 hospital release notes were analyzed. 51.1% of subjects (n=47) were aged between 21 and 30 years, 34.78% (n=32) were aged between 31 and 40, 10.86 % (n=10) under 20, and 9.38 (n=3) over 41 years (Figure 1). The urban/rural distribution was approximately equal (Figure 2), with an insignificantly increased percentage in the case of patients from rural areas.

### Clinical data

All the analyzed subjects were pregnant, 15 with a pregnancy of less than 36 weeks (16.30%), 2 ectopic pregnancies and 2 abortions, and the rest of the patients with pregnancies between 37 and 41 weeks. 44.57% received anticoagulant treatment with LMWHep (Table 1). The reasons for approaching this therapy are specified in Table 2, the most frequent being the care given to the mother for the uterine scar due to a previous surgery (41.5%). 34. 78% (n=32) gave birth naturally and 46.74% by caesarean intervention. 8 cases of death of fetus/newborns were registered without causal relationship between the administration of LMWHep and the recorded data. However, most newborns presented a very good Apgar index (70.65%).

**Table 1.** Characteristics of subjects according to their discharge note

	**Diagnostic**		**LMWHep treatment**	**The type of birth**		**Apgar index**		**Aborted/dead**
	
	**Pregnancy < 36 weeks**	**Pregnancy > 36 weeks**		**N**	**C**	**0–7**	**8–10**	
Patients (n)	15	73	41	32	43	17	65.00	8
Patients (% of total)	16.30	79.35	44.57	34.78	46.74	18.48	70.65	16.30

N-natural birth, C-caesarian intervention

Data of laboratory analyzes highlight good hemoglobin reports (only 7.32% presented values below normal values). 31.7% of subjects presented a hematocrit value under 35%, and all the patients had fibrinogen percentage higher 400 mg/mL.

### Cell Viability Assessment

**Fig. 3.**
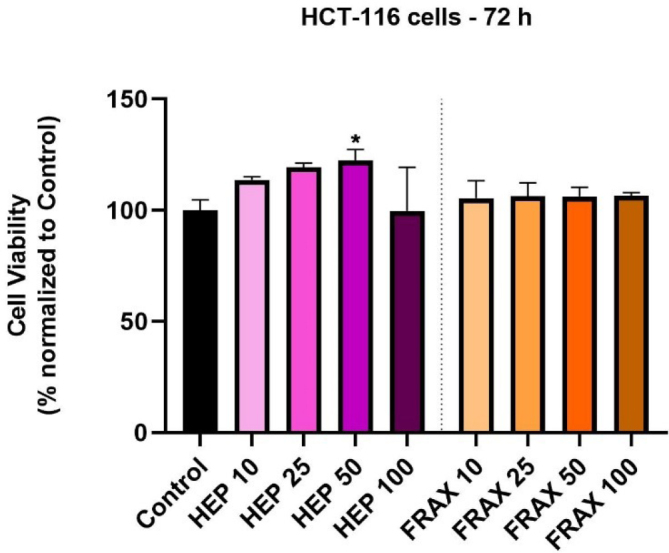
In vitro HCT 116 cell evaluation after 72 h of incubation with HEP and FRAX (10, 25, 50, and 100 UI) by performing the MTT assay. The data are presented as viability % normalized to untreated cells (expressed as average values ± SD of three independent experiments). One-way ANOVA analysis and the Dunett’s multiple comparisons post-test were conducted to identify the statistical differences between obtained data (* p < 0.1).

To evaluate the capacity of HEP and FRAX to interfere with the cellular viability of HCT 116, cells were treated with 4 concentrations from each substance (10, 25, 50, and 100 UI), for 72h, then the MTT assay was applied. Obtained results show a slight stimulated effect up to the concentration of 50 UI (up to 120%) in case of HEP, and a gentle inhibitory effect at 100 UI (90%). When about FRAX, similar results were obtained, however with an attempt at more observable linearity. Thus, at all tested concentrations, the viability was approximately like that observed in the case of untreated control cells (around 100%) (Figure 3).

### Cell Morphology and Confluence

**Fig. 4.**
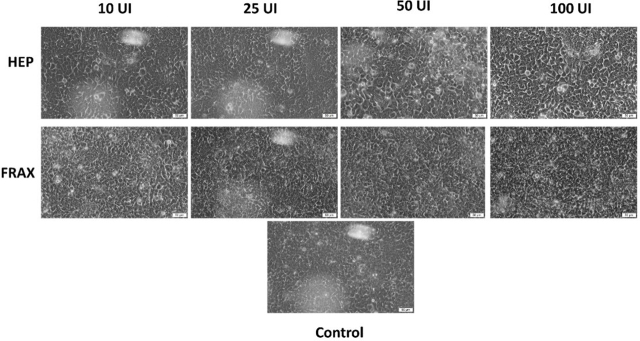
Images showing the confluence and morphological aspect of HCT 116 colorectal carcinoma cells after 72 h of treatment with HEP and FRAX at 10, 25, 50 and 100 UI. The scale bars represent 50 µm.

The next step of the present study was the microscopic examination of the HCT 116 cells treated with the four concentrations of HEP and FRAX. Results are depicted in Figure 4. It was observed no significant modification in morphology or confluence of cells, data that are consistent with those obtained in the case of viability assay. However, in the case of HEP 100, the cells seem not to be as crowded as in the other cases, thus a more distinct shape is observed, with the slight elongation specific to this cell line (Figure 4).

### Wound Healing Assay

**Fig. 5.**
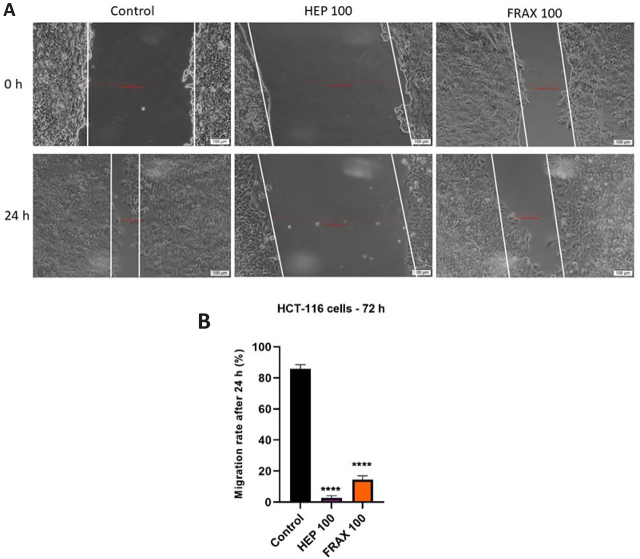
A) Pictures illustrating the migratory capacity of HCT 116 cells after treatment with HEP 100UI and FRAX 100UI for 24h. The scale bars represent 50 µm. B) Graphic depiction of the migratory potential of HCT 116 cells after the treatment with HEP 100UI and FRAX 100UI. The bar graphs are exposed as % of wound closure after 24 hours compared to the initial distance at t0. The results are expressed as averages ± SD of 3 independent experiments. One-way ANOVA analysis and the Dunett’s multiple comparisons post-test were applied to follow the statistical differences between obtained results (**** p < 0.0001).

To assess the impact of HEP and FRAX on the migratory properties of colorectal carcinoma cells, a wound healing assay was applied. HCT 116 cells were treated with the highest concentration (100 UI) of each substance. The results were depended on the tested HEP, the most potent effect being observed in case of HEP 100, with wound healing rates of 2,6%, significantly more potent than in case of control, where the wound healing rates was of 85,9% (Figure 5). When about LMWHep, these manifested also a good inhibitory property of the cell migration, with a wound healing rate of 14.52%.

### Nuclear Morphology Evaluation

**Fig. 6.**
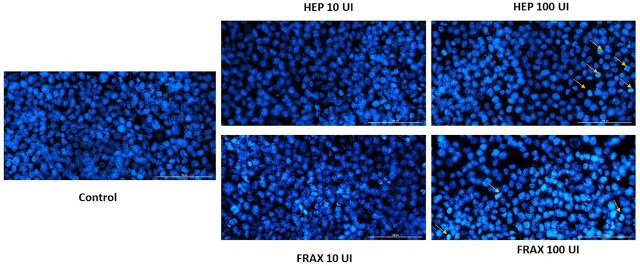
Pictures of the HCT 116 cellular nuclei counterstained with Hoechst 33342 reagent after 72 h of treatment with HEP and FRAX 10 and 100UI. The yellow arrows assign nuclei with apoptotic signs. The scale bars represent 100 µm.

The last step of the in vitro study was the analysis of nuclear morphology, in order to identify if the cellular death occurred by necrosis or apoptosis. For this experiment, two concentrations were tested: 10 and 100 UI of each type of HEP. Hoechst reagent was used to counterstain the cell nuclei, and treated cells were compared with unstimulated (control) cells. In the case of the lowest concentration, no significant changes were observed, the nuclei present a round and regular form, without signs of fragmentation. Instead, in case of the highest concentration, signs of apoptosis were observed. HEP 100 induced membrane blebbing, nuclear condensation and fragmentation (highlighted with the yellow arrows in Figure 6). FRAX 100 also induced chromatin condensation and nuclei fragmentation (Figure 6).

## Discussions

It is widely acknowledged that the status of pregnancy leads to the downregulation of the hemostatic system, resulting in a hypercoagulable state. These physiological changes are crucial in minimizing the likelihood of blood loss during childbirth, however, they also increase the risk of thromboembolism both during pregnancy and after delivery [24]. The pro-coagulant state could also stimulate the appearance of gestational vascular complications as placental abruption, recurrent early and late miscarriage, fetal growth restriction, intrauterine death, and stillbirth. This is particularly evident in cases of obtained or inherited thrombophilia [25,26]. Thereby, HEP and LMWHep are anticoagulant medications commonly used during pregnancy to prevent or treat blood clotting disorders. These medications are essential in managing conditions like deep vein thrombosis, pulmonary embolism, and certain autoimmune disorders that increase the risk of blood clots. HEP does not cross the placental barrier significantly, making it a safer option for pregnant women compared to some other anticoagulants. LMWHep has a good safety profile during pregnancy, and it is considered a category B drug by the United States Food and Drug Administration (FDA). This marks that studies on animals have not shown dangers to the fetus, [27]. The objectives of the present study was to analyze and interpret the data related to the consumption of HEP or its derivates in the case of pregnant women and to test them on colorectal carcinoma.

Maternal age is a critical factor influencing reproductive health and pregnancy outcomes. Advancements in healthcare and societal shifts have led to variations in the average maternal age at childbirth. Advanced maternal age, commonly defined as age 35 and above, is associated with a gradual decline in female fertility due to factors such as decreased ovarian reserve and altered hormonal profiles. This decline may lead to prolonged time-to-pregnancy and other different healthcare problems as preeclampsia, congenital anomalies, low birth weight, gestational diabetes mellitus, and premature birth [28]. In the present study, 86.7% of the women were aged up to 35 years.

Analyzing the discharge files taken in account in the present study, it was found that, 44.57% received anticoagulant treatment with LMWHep. The safety profile of LMWHep was discussed in different studies [29, 30]. One of researchers analyzed the incidence rates of different pregnancy complications that could appear (LMWH group vs control untreated group). Results showed 8.7% / 6.5% for preterm delivery, 1.56% / 1.1% for thrombocytopenia, 0.7% / 0.3% for stillbirth, 2.7% / 2.2% for fetal growth restriction, 1.4% / 1.0 % for severe preeclampsia, and 10.7% / 7.8% for antenatal bleeding. The rate of bleeding during delivery and the caesarean section rate were similar in the two groups. The risk of major bleeding during labor (>1000 ml) was not different in the 2 groups [31]. Thus, LMWHep was considered safe for administration in case of pregnant women. The main cause of LMWHep administration in this study was care given to the mother for the uterine scar due to a previous surgery (41.46%), followed by medical induction of labor failure (14.63%). Nevertheless, most newborns had a good Apgar index (Table 1). 97,6% (n=40) of patients treated with anticoagulant gave birth by caesarean section. Following hemoglobin values, it is observed that only 7.3% present a lower level than the inferior limit, thereby 31,71% presented a hematocrit level < 35%, which may be due to the surgical intervention, although the values are kept close to the allowed limit. A study highlighted that a high hemoglobin concentrations is associated with high birth weight and low hemoglobin level being associated with low length of newborns [32]. Analyzing the fibrinogen values, it was observed that 100% of the subjects had slightly increased values (Table 2). Postpartum hemorrhage, which accounts for a significant number of maternal fatalities globally, is responsible for nearly sixty thousand deaths annually. The role of fibrinogen in bleeding and coagulation is noteworthy. Research indicates that decreased levels of fibrinogen prior to childbirth can serve as a predictive factor for severe hemorrhage. Thus, fibrinogen concentrate therapy might be very useful, and slightly increased values present an advantage in this case [33].

**Table 2.** Diagnostic and laboratory analyzes according to the discharge ticket

		**Patients (n)**	**Patients (% of total)**
Discharge diagnosis	Care given to the mother for the uterine scar due to a previous surgery	17	41.46
	Medical induction of labor failure	6	14.63
	Birth by caesarean section	2	4.88
	Other diagnosis*	15	36.59

Hemoglobin value	Under 12 g/dL	3	7.32
	Over 16 g/dL	32	78.05

Hematocrit value	Under 35 %	13	31.71
	Over 46%	0	0.00

Coagulation/fibrinogen value	Under 200 mg/dL	0	0.00
	Over 400 mg/dL	41	100.00

Other diagnostics* include: tubal pregnancy (n=1), care given to the mother for the insufficient growth of the fetus (n=1), premature detachment of the placenta (n=1), severe or delayed hemorrhage following abortion and ectopic and molar pregnancy (n=1), premature rupture of the membranes, with the onset of labor in 24 h (n=1), care given to the mother for a disproportion due to an abnormally large fetus (n=1), care given to the mother for a pelvic presentation (n=1), maternal care for fetal injuries resulting from a viral disease (n=1), labor and birth complicated by unspecified fetal distress (n=1), care given to the mother for the intrauterine death of the fetus (n=1), gestational hypertension without specific proteinuria (n=1), physiological lause (n=1), hereditary hemorrhagic telangiectasia (n=1), maternal care for signs of fetal hypoxia (n=1), and partial retention of the placenta and membranes, without hemorrhage (n=1.)

Cancer and pregnancy are other sensitive subjects, especially because it can endanger both the life of the mother and the fetus, and existing treatments are limited, especially due to high toxicity. CRC in pregnancy is uncommon but not rare [34], and the incipient symptoms of the malignancy superpose with common physiological modifications encountered in pregnant women and can trippingly be dismissed. In addition, the management of cancer in pregnancy elevates medicolegal and ethical issues, and narrow documented solutions exist to guide a management approach.

In vitro tests show a stimulation of cell growth, when HEP and FRAX were tested on the HCT 116 cell line, without obvious morphological or confluence changes. Similar results were obtained by Chatzinikolaou et al., where HEP at 100 µg/mL stimulated cell proliferation of HT-29, HCT 116, and SW1116 [35]. The study highlighted that HEP was capable to modulate expression of genes implicated in cell cycle regulation through upregulation of p38 MAP kinase to stimulate colon cancer cell growth [35]. Another study obtained similar results, HEP and enoxaparin at 100 µg/mL showed a significant increase in HCT 116 cell viability (p < 0.001), after 24h of incubation. In addition, HEP and LMWHep respectively, didn’t manifested any effect on the proliferation potential of endothelial cells [36], primary human osteoblasts, and human pulmonary epithelial cells (A-549 cell line) [37,38]. Thereupon, heparins seem to have no significant effect on the viability of cancer cell lines [39,38], this being expected, as HEP is an endogenous composite encountered in human body. Instead, these compounds are well-known to manifest anticancer and antimetastatic potential in vivo, but because of other mechanisms than antiproliferative or cytotoxic effects [40]. A study followed by Jayson and Gallagher on CaCo-2 colorectal adenocarcinoma showed that the length of an oligosaccharide print its capacity to obstruct the potential of fibroblast growth factor [41].

While HEP and its derivatives are able to help patients with tumors as anticoagulants, they can also directly impact tumor advancement via metastatic inhibition. Different studies support the idea that blood-borne metastasis are meaningfully made easier by interactions between blood platelets and disseminating tumor cells [42,43,44]. Interaction between anticoagulants or antiplatelet agents with tumor cells strongly downregulates both experimental and spontaneous metastasis. Additionally, it has been noted that a reduced level of circulating platelet is associated with a decline in distant metastases [45]. The interaction between platelet and tumor cells serves as a protective mechanism against immune surveillance. Additionally, platelets have the ability to release essential growth factors and cytokines upon activation, thereby providing cancer cells with crucial stimuli for growth [46]. Furthermore, the metastasis of tumor cells in mice is facilitated by vascular cell adhesion molecules, such as P- and L-selectins, through their interaction with endothelium, platelets, and leukocytes. Tumor invasion and metastasis are further promoted by the degradation of heparan sulfate in the extracellular matrix by heparanase. A new study identified a non-anticoagulant species of HEP that specifically inhibits heparanase enzymatic activity, selectin-mediated cell-cell interactions or both. As much, it was observed that specific inhibition of heparanase with heparin derivatives or selectin interactions in mouse models of MC-38 colon carcinoma and B16-BL6 melanoma reduces metastasis [47]. The inhibition of angiogenesis is another crucial therapeutic approach for the treatment of cancer, as this process plays a significant role in tumor progression. Recently, there has been significant progress in the development of heparin-based angiogenesis inhibitors, which are considered a promising class of therapeutics in the clinical setting. One such inhibitor, known as Taurocholate conjugated LMWHep derivative (LHT7), has undergone testing and demonstrated a remarkable potential as a potent and multi-targeting angiogenesis inhibitor against various angiogenic tumors [48].

In vitro scratch assays are performed to examine cellular migration. The obtained data in the present work highlights a significant potential of HEP and FRAX to reduce cell migration (Figure 5). A recent study pointed out that LMWHep can ameliorate the three-month and six-month survival rate of patients with cancer. Firstly, it was considered that HEP’s anti-metastatic potential was manifested via antithrombotic pathways, but more recent findings suggest that the anti-cancer strength mull over an independent property [16]. In another research it was observed that epidermal growth factor receptor tyrosine kinase inhibitor ZD1839 downregulates proliferation of colorectal cancer cells of patients with metastatic colorectal cancer forms. ZD1839 has the potential to up-regulate p27(Kip1) and induce apoptosis in a specific group of patients with colorectal cancer [49], different signs of apoptosis being observed in the present study too, after performing the Hoechst assay. Recent research highlighted that a heparan sulfate mimetic PG545 exhibits a potent anti-lymphoma effect and manifest only gentle anticoagulant activity. After analyzing the pro-apoptotic effect of PG545, results revealed that this elicits apoptosis, inducing endoplasmic reticulum stress and autophagy via activating the NFκB pathway [50]. In another research, Yin et al. analyzed the combination of LMWHep and adriamycin treatment to adriamycin alone. The lung metastasis of breast cancer cells in C3H mice was found to be downregulated upon administration of the combined therapy. Additionally, it was observed that HEP inhibited the expression of vascular endothelial growth factor in tumor tissue, thereby inducing apoptosis in cancer cells [51]. Another study analyzed the synergism and observed that intraperitoneal tumor growth in rats receiving intraperitoneal application of taurolidine and combined taurolidine/heparin was significantly diminished when compared to the control group [52].

## Conclusions

HEP and LMWHep are frequently used in case of pregnant women, because to their physiological downregulations of the hemostatic system into a hypercoagulable state, posing elevated potential to develop coagulation problems, that have to be managed. In the present study, 92 discharge tickets of pregnant patients admitted to the “Pius Brînzeu” hospital in Timișoara were analyzed. The results of the analysis highlighted the fact that 42 patients were treated with anticoagulant medication, and the most frequent cause of administration was the care given to the mother for the uterine scar due to previous surgery, without side effects, and with a good Apgar index observed in newborns. When tested on cancer cells, HEP and LMWHep manifested significant anti-migratory effect and good pro-apoptotic potential, suggesting the possible potential use in the case of colon cancer, especially in the segment of pregnant patients, for whom the current medication is limited. According to these findings, future studies are required to confirm the applicability of HEP and its derivatives in the treatment of these particular cancer category of patients.
